# Stone Throwing as a Sexual Display in Wild Female Bearded Capuchin Monkeys, *Sapajus libidinosus*

**DOI:** 10.1371/journal.pone.0079535

**Published:** 2013-11-21

**Authors:** Tiago Falótico, Eduardo B. Ottoni

**Affiliations:** Institute of Psychology—University of São Paulo, São Paulo, Brazil; University of Oxford, United Kingdom

## Abstract

Capuchin monkeys (*Sapajus* spp.) in captive settings frequently manipulate and throw objects. In the wild, they may push or drop stones and sticks toward targets during inter- or intraspecific threat displays. In addition, female capuchin monkeys exhibit a broad repertoire of behaviors during their proceptive period, including facial expressions, vocalizations, stereotyped body postures, and touch-and-run behavior. This study reports stone throwing as a newly-described communicative behavior during the proceptive display of females in a group of bearded capuchin monkeys (*S. libidinosus*) in Serra da Capivara National Park, Brazil. During a two-year study, three females from one group were seen throwing stones at males during their proceptive phase. After this period, three other females in the same group exhibited the same behavior. Although it may be possible that this pattern is the result of several independent innovations by each female, the apparent absence of this behavior in other groups leads us to suggest that we have documented the diffusion of a new behavioral trait or tradition within this capuchin social group.

## Introduction

Aimed throwing behavior requires a series of coordinated movements, associated with adjusting the angle and timing of an object's release in order to successfully hit the target [1]–[4]. Aimed throwing probably played an important role in the aggressive and defensive behavior of protohominids against predators and cospecifics, and certainly did so in the subsequently in hunting [5], [6], leading to the development of tool-using technologies such as projectiles and spears [3], [7]. Selection for object throwing capabilities during human evolution (of great importance to allow defense and hunting) may have led to neural structures including time sequencers (to control the release of objects) and enlarged areas of the brain - such as the motor cortex, which has been speculated to secondarily have been used for fine manual gestures, tool manufacture, and even language processing [1], [8], [9]. Moreover, accurate throwing behavior in humans may have been an important adaptation for hunting during human evolution (perhaps around 2My ago [5]), initially involved in the throwing of natural objects and later in the throwing of hunting tools such as spears and javelins [7].

Capuchin monkeys (*Cebus* spp and *Sapajus* spp – the latter one previously referred to as the tufted species of the genus *Cebus*) are New World monkeys characterized by high brain/body size ratios and enhanced manipulative abilities and manual dexterity that enable them to solve problems in a wide range of manipulative tasks [10]. They also are reported to have social traditions in foraging and affiliative behavior [11]–[13].

In captivity, there are many reports of spontaneous object throwing, but most are anecdotal and lack detailed behavioral descriptions [14]–[16]. There are, though, reports of experimental situations, like Westergaard et al [17], that reported that capuchin monkeys (*Sapajus* sp) threw food as a means to share it with other monkeys housed in cages 1 m apart. Moreover, in an experimental situation, capuchin monkeys (*Sapajus* sp) successfully used stones to solve a foraging problem throwing stones into a syrup container and then retrieving them - coated with syrup. Over 83% of these episodes involved overarm throws, and success was positively correlated with individual hand preference. Sex based differences in throwing accuracy were not found [18]. In a stone throwing task that required tufted capuchins to select and throw a stone toward a bucket containing food, Cleveland et al. [19] found that individuals exhibited a strong preference for stones of a given mass, performing better when using stones of the preferred mass. Finally, in an experiment designed to test the capuchins' understanding of the difference between functionally appropriate or inappropriate tools, Evans and Westergaard [20] reported that the monkeys choose the appropriate throwing tools more frequently to recover food (i.e. a projectile attached to a line) than an inappropriate tool (i.e. a non-attached projectile, a projectile attached to shorter line, a projectile without line or a line without a projectile tool).

Capuchin monkeys have been observed pushing or dropping stones and sticks in inter- and intraspecific threat episodes, both in captivity [14], [21] and in the wild [22]–[24; personal observation]. In these cases, though, the release is rarely aimed, the movement is not ballistic, and the object usually ends up far from the threat display target. Stone banging to enhance a threat display was also reported [25], but there are no reports of aimed throwing by wild capuchin monkeys.

In the case of apes, there is evidence that some species use tools for communication purposes in the wild, including sexual displays. Male chimpanzees of Mahale, Tanzania, use leaf-clipping to attract females during courtship [26], while those in the Ngogo community (Kibale National Park, Uganda) use both leaf-clipping and branch-waving for the same purpose [27]. Chimpanzees were also described throwing stones in charging displays and during play, and also throwing rocks into streams, thereby producing loud splash sounds that intimidate others [28]. Orangutans have been seen to use branches to hit other animals [29]. This involved the use of sticks or branches agitated or thrown at the other individual.

Female capuchins exhibit a broad repertoire of behaviors during the proceptive phase, including facial expressions, vocalizations, stereotyped bodily postures, and touch-and-run behavior - where the female touches, grabs, or pulls a part of the male's body and then retreats. These behavioral changes are external manifestations of sexual proceptivity and subsequent receptivity [30]. Initially, the male may ignore the female displays and avoid her. On other occasions, he may threaten and briefly chase the female, especially in response to touch-and-run behavior. Later, the male may respond with similar facial and bodily displays leading to copulation. During a female's proceptive period (estimated from 1 to 7 days, depending on the study [10], she follows a male (usually the dominant male) and directs most of her solicitations to him [10], [31]. If the female is not impregnated, the cycle restarts after +/−21 days [10].

In this study we describe stone throwing as a communicative behavior exhibited as part of the proceptive display in female *Sapajus libidinosus* inhabiting Serra da Capivara National Park (SCNP) in Northeastern Brazil. Although most tool use in this population is foraging-related, we describe here the use of stone throwing as a socio-sexual behavior. We observed this behavior in only one of the two groups we studied in the park. This group has been studied for 4 years. In two other groups of *S. libidinosus* studied in SCNP by Mannu and Ottoni [32] for 2 years, stone throwing as a courtship behavior has not been reported. Bearded capuchin monkeys at SCNP exhibit a unique tool-kit. In addition to the use of “hammer” stones to crack hard fruits or seeds, often observed in savannah and dry bush populations [13], all studied groups in SCNP frequently use stones as “hammer” to loosen the soil for digging, and sometimes as a “hoe” to cut plant parts and pull the soil when digging. They also use stones as “hammers” to pulverize or dislodge rocks, and sticks as probes to remove prey such as lizards and bees from cavities [32], [33].

## Methods

### Location

The study was conducted at the Serra da Capivara National Park (SCNP), in Piauí state, northeastern Brazil. The SCNP area is classified as a geoclimatic domain called *Caatinga*: a mosaic of xerophytic vegetation with patches of deciduous forest along the narrow, more humid valleys surrounded by high cliffs within a semi-arid climate. The study area is located in the southeastern border of the park (limiting coordinates: North: 8°49′S, 42°33′W; South: 8°51′S, 42°33′W; East: 8°50′S, 42°32′W; West: 8°50′S, 42°34′W). Capuchins at SCNP obtain most of their food by exploiting naturally occurring resources. They are provisioned in the dry months of July to November by the park staff (about three dozen bananas every two days), and spent less than an hour per day eating the provided food.

### Study groups

We observed two partially sympatric groups. The Pedra Furada (PF) group was composed of 45 individuals (8 adult males and 16 adult females) at the beginning of the study, and the Bocão (BC) group was composed of 27 individuals (4 adult males and 7 adult females). These groups occasionally met and foraged together for minutes or hours, usually without conflict (occasional agonistic episodes occurred between particular individuals, but this never involved conflict between whole groups).

The groups were systematically followed for 20 days per month, from initial contact early in the morning to the end of the day (or until contact was lost). Data on PF group were collected for 23 months (Sep/2007–Jul/2009, total of 226 days and 38 of female proceptive periods), and on BC group, for 12 months (Mar/2008–Feb/2009, total of 99 days and 10 of female proceptive periods). We also collected data (only on PF group) during a subsequent 5-day' visit to the research site (∼10 hours of observation from 03 to 07/Jul/2012).

### Data collection

During data collection (part of a study on tool use), we recorded all observed occurrences of tool use behavior. The number of researchers observing a capuchin group during each sampling day was two, TF and a field assistant.

We followed Shumaker et al.'s widely accepted definition for tool use: “the external employment of an unattached or manipulable attached environmental object to alter more efficiently the form, position, or condition of another object, another organism, or the user itself, when the user holds and directly manipulates the tool during or prior to use and is responsible for the proper and effective orientation of the tool” [16].

Stone throwing episodes by females were noted using audio and/or video recording. The female and the target male in each episode were identified. Each throwing event was scored and a series of such events was considered a single episode when they occurred between the same individuals, in the same location and less than 10 min apart. We considered a hit when the thrown stone touched the body of the male before contacting the ground. All proceptive periods were inferred by the occurrence of soliciting behavior by the females.

The stones used as tools (in this and any other contexts), when possible, were collected, measured and weighed. Length was measured along the longest axis of the stone. Width was measured as the average of three equidistant perpendicular measures to that axis along the widest side of the center of the stone. Thickness was determined by the average of three measures taken perpendicularly to the three width measures.

The research in Serra da Capivara National Park was exclusively observational and the researchers had only visual contact with the monkeys. The research was previously approved by federal environmental agencies IBAMA/ICMBio (authorizations 037/2007/DIREC and 14825-1), and adhered to the laws governing animal research in Brazil, the American Society of Primatologists' principles for the ethical treatment of primates, and followed all ethical guidelines for animal research of the Institute of Psychology-USP.

## Results

**Figure 1:**
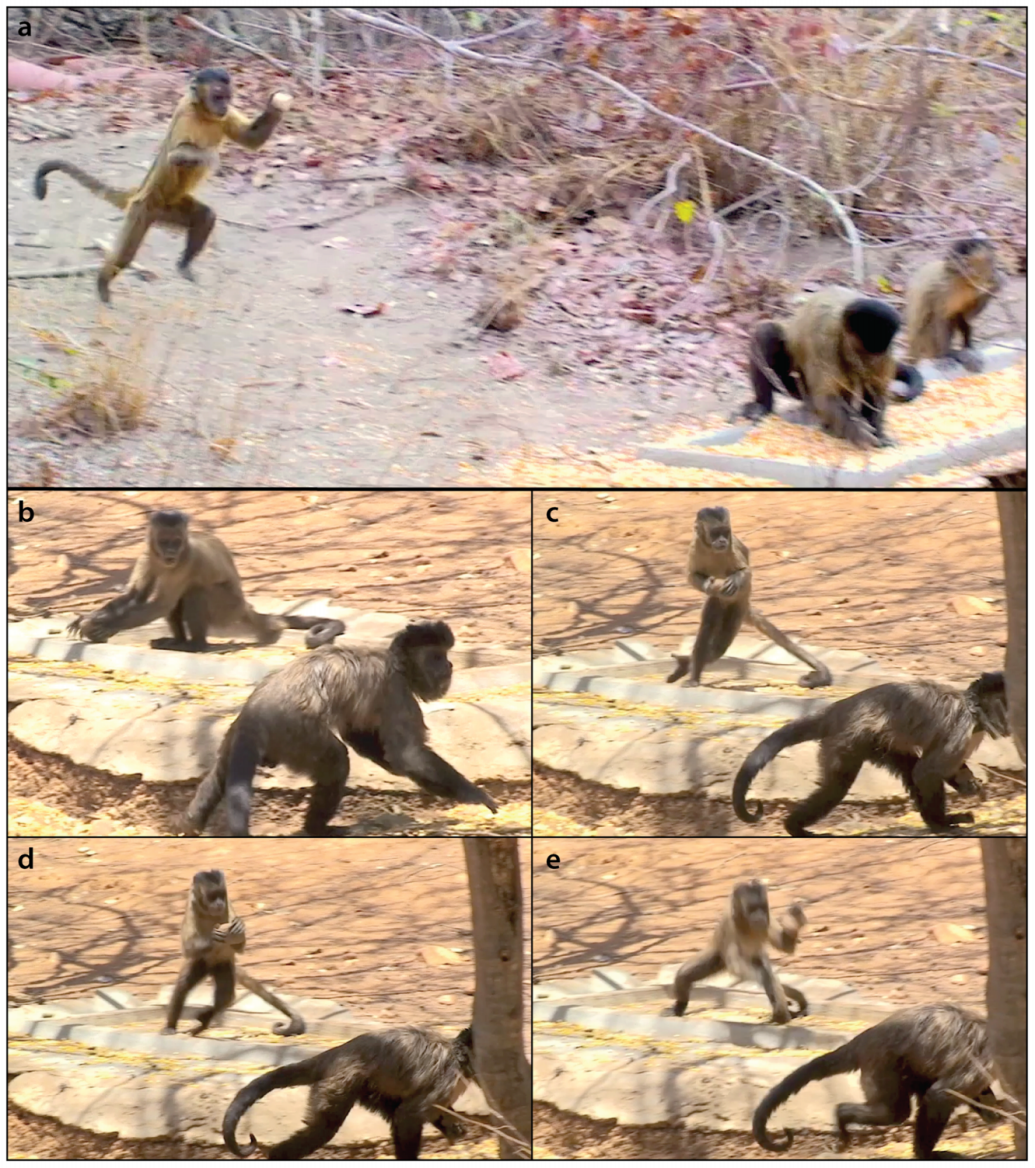
Stills from video recordings, showing moments of two throwing events. (a) Pedrita running with a stone just before throwing it at Beiçola;(b) Pedrita picking up a stone, (c, d) running, and (e) throwing the stone at Bochechudo. The video is available as supplementary material - Video S1.

During a total contact time of 1290.2 h with PF group and 426.3 h with BC group, we recorded 49 proceptive periods for at least 17 of the 23 adult females in the groups (Table 1). During 28.5% of contact time, one or more females in our study groups were in estrus (PF: 385.1 h; BC: 106.2 h). Three of 13 females in the PF group (but none in the BC group) were seen throwing stones at males during their proceptive phases. All throwing postures were overarm: the females held the stones at or above shoulder level before throwing it (Figure 1 and Video S1). They usually stood bipedally while throwing, but there were cases (not quantified) of throwing using a tripedal stance. The target males were always high-ranking individuals, but not always the alpha males (who were the targets in 22.2% events). Low-ranking males were never targets of stone throwing.

**Table 1:** Proceptive periods recorded for the capuchin monkeys of PF and BC groups from Serra da Capivara National Park.

Group	Female	Proceptive phases recorded	Proceptive phases with throwing events	Throwing events recorded
**PF**	Pedrita	2	2	32
	Benne	1	1	8
	Ninfa	2	1	2
	Canela	9	0	-
	Jurema	2	0	-
	Maçã	4	0	-
	Romã	2	0	-
	Lica	3	0	-
	Diana	2	0	-
	Tatu	1	0	-
	Encrenqueira	4	0	-
	Gorda	2	0	-
	Alice	1	0	-
	Unidentified	3	0	-
**BC**	Elvira	1	0	-
	Perninha	5	0	-
	Cássia	1	0	-
	Tara	3	0	-
	**TOTAL**	**48**	**4**	**52**

We treated all the throwing events by the same female at the same location and less than 10 min apart as a single throwing event.

When hit by a stone (N = 10), the males looked towards the female and, on two occasions, briefly threatened and chased her. This behavior response could be exhibited even if when the stone hit the ground near him (<1 m,). After that, they usually resumed their ongoing activities. The threats were not very severe and the male's response was not different from when a female grabbed the male's tail during touch-and-run behavior.

No female was observed throwing stones outside of her proceptive period. The three females from PF group that exhibited throwing behavior did so at different frequencies (Table 2). Pedrita was the most frequent thrower (76.2%). She was observed on 5 days during 2 proceptive periods. She threw stones at 2 different males.

**Table 2:** Throwing events by capuchin monkey females of PF group.

Female	Date	Throwing events	Target male	Hits
**Pedrita** *	26/09/2007	8	Beiçola	1
	28/09/2007	3	Beiçola	2
	12/11/2007	9	Bochechudo	1
	13/11/2007	12	Bochechudo	2
**Benne**	17/10/2007	4	Bochechudo	0
	18/10/2007	4	Bochechudo	0
**Ninfa**	01/10/2009	2	Torto	2
	**TOTAL**	**42**		

* Died in 01/2008.

Hits were considered when the thrown object hit the male before reaching the ground.

In Table 3 we present data collected between 01/2012 and 08/2012 for the PF group, using the same methods as in the previous study period. We found that 3 additional females in the group were also performing stone throwing behavior during their proceptive period. Two of these females were immatures during the original study (Vesga - 8 events and Alice - 13 events), and the other was an adult during the 2007–2009 study period (Gorda - 1 event), but was not seen throwing stones in either of the two proceptive periods we observed (Table 1).

**Table 3:** Throwing events by female capuchin monkeys of PF group after the original research period (01/2012–07/2012).

Female	Date	Throwing events	Target male	Hits
**Gorda**	06/07/2012	1	Torto	0
**Vesga**	06/2012+	1	?	?
	06/07/2012	1	Torto	1
	06/07/2012	6	Zandor	1
**Alice**	23/07/2012*	13	Torto	?
	**TOTAL**	**21**		

+ Camila Coelho, pers. comm.

* Raphael Cardoso, pers. comm.

These events were recorded during visits to the groups by the authors, or by other researchers working with this group.

In 2007–2008, we collected eight stones used as projectiles by two of the females (Pedrita and Ninfa). We only collected stones that we were able to identify were thrown by a female. The average weight of the stones was 52.0 g (+/−27.1 - range 19–84), and the average length, width and thickness were, respectively, 4.6 cm (+/−0.9 - range: 3.6–5.9), 3.1 cm (+/−0.52 - range: 2.1–3.8) and 2.2 cm (+/−0.64 - range: 1.1–3.1).

## Discussion

The sexual display of female capuchin monkeys is a very conspicuous behavior. It has been described in *Sapajus* spp., *C. capucinus* and *C. albifrons*, and varies in form from a simple display in the two *Cebus* species to very elaborate and extensive display in *Sapajus* spp. While *C. capucinus* do not present a high variety of displays, it includes a unique facial expression described as duck face in which monkeys protrude their lips during sexual display [34]. A duck face display has not been reported in *Sapajus* spp., but this genus appears to include the species that have the most diverse sexual displays [10]. The incorporation of stone throwing, which has been reported only in *S. libidinosus*, further suggests that among tufted capuchins there is a great plasticity in display behaviors.

We have studied 4 groups of *S. libidinosus* at SCNP over the course of 5 years, however, only females of PF group have been observed throwing stones [32], [35]. Moreover, during a 5-day field trip to SCPN in July/2012, we observed stone throwing by two other adult females from PF group during their proceptive period. Gorda was observed throwing a stone at Torto once, and Vesga (a juvenile not observed in estrus in the original period of research) was seen throwing stones 7 times (2 hits) on 2 days of observed estrus. Vesga also was seen throwing stones during one of her proceptive periods, on June/2012 [Camila Coelho, pers. comm.], and, in July/2012, another female (Alice) exhibited the throwing behavior (N = 13) during her proceptive period [Raphael Cardoso, pers. comm.]. In each case, the male hit with the stone copulated with the sexually displaying female. We have no data on whether stone throwing has any effect on a female's success in soliciting copulations from an adult male (e.g. decreasing the male latency to mate).

All adult individuals in our study groups (even in the groups in which we did not observe females' stone throwing) are proficient at using stones (and other objects) as tools in various contexts [35, Falótico & Ottoni, unpublished data]. In this regard, it is possible that some females of PF group could have independently added stone throwing to their sexual display.

The only similar observation is a recent report from captives in a zoo [36]: in that study a single female *S. libidinosus* threw stones (N = 6) at males during estrus, in a similar manner to the cases reported here. This could indicate that stone throwing during sexual solicitation is more widespread in *S. libidinosus* than previously thought or is a commonly “reinvented” behavioral pattern. In our study groups, we believe that the possibility that 6 females in the same group independently added stone throwing to their sexual display is unlikely, since females from other groups in SCNP have access to stones, use stones in foraging contexts, but were never observed throwing stones during sexual displays. In addition, Mannu and Ottoni [32] studied two groups of bearded capuchins in the same area over a two year period and did not observe stone throwing in this context, nor has this behavior been reported in another long-term study site where stone tool use by bearded capuchins is common, Fazenda Boa Vista [37], [38].

One possible explanation for the presence of this behavior in the repertoire of several females in PF is that this behavior was disseminated through social influences. Although stone throwing was not very frequent, occurring at an average rate of 7.8 times per throwing female proceptive period, non-throwing females could observe it performed by others on several occasions. The pattern of occurrence of the behavior, restricted to just one group and with an increasing number of females performing it, suggests that social learning played an important role in behavioral transmission. That is, the behavior was independently ‘invented’ by one or more females, and then copied by others as a form of “enhanced” display. It is not possible to determine when this behavior appeared in PF, but it was already exhibited by some females from the beginning of our study, and appears to be slowly spreading in the group: we first observed only 3 of 14 individual females exhibit this behavior (Table 1), and 3 years after the original research, an additional 3 females (1 who was and 2 who were not adults during our original study) were seen throwing stones at males.

If the social learning hypothesis is correct, this behavior is likely to remain limited to its original group, since *S. libidinosus* females are strongly philopatric, and seldom leave their natal group [39]. Moreover, during intergroup encounters, males from neighboring groups were seen to interact, whereas adult females appeared to remain apart and avoid contact.

It's also important to note that most tool use by capuchins (and primates in general) is foraging-related [10], and is a predominantly male activity [35], [37], [40], [41, Falótico & Ottoni, unpublished data]. As a communicative behavior, it is reminiscent of object percussion as a male sexual signaling [42] or threat display [24] in capuchins, as well as the leaf-clipping behavior by Mahale male chimpanzees reported by Nishida [26].

Continuing observation of the PF group and other nearby groups will help us determine whether the stone throwing behavior will spread to more or all adult females in the PF group, and eventually appear in other groups - either by independent invention or diffusion by a rare migrating female. We have perhaps, a unique opportunity to document the early phase of the diffusion of a new behavior, whose potential future dissemination history we are looking forward to following.

## Supporting Information

Video S1 **Stone throwing as sexual display by female capuchin monkeys.** The identity of the female and male involved in each event is displayed in the video subtitles.
(MP4)
